# The P300 event related potential predicts phonological working memory skills in school-aged children

**DOI:** 10.3389/fpsyg.2022.918046

**Published:** 2022-10-12

**Authors:** Vanessa Harwood, Daniel Kleinman, Gavino Puggioni, Alisa Baron

**Affiliations:** ^1^Department of Communicative Disorders, University of Rhode Island, Kingston, RI, United States; ^2^Haskins Laboratories, New Haven, CT, United States; ^3^Department of Statistics, University of Rhode Island, Kingston, RI, United States

**Keywords:** electrophysiology, P300, children, phonological working memory, language

## Abstract

The P300 event related potential (ERP) has been cited as a marker of phonological working memory (PWM); however, little is known regarding its relationship to behavioral PWM skills in early school-aged children. The current study investigates the P300 ERP recorded in response to native and non-native (English and Spanish) phoneme contrasts as a predictor of PWM skills in monolingual English-speaking first and second grade children. Thirty-three typically developing children, ages 6–9, completed a battery of phonological processing, language, and cognitive assessments. ERPs were recorded within an auditory oddball paradigm in response to both English phoneme contrasts (/ta/, /pa/) and Spanish contrasts (/t̪a/, /d̪a/). The P300 ERP recorded in response to English phoneme contrasts significantly predicted standard scores on the Nonword Repetition subtest of the Comprehensive Test of Phonological Processing, Second Edition. Spanish contrasts did not elicit a P300 response, nor were amplitude or latency values within the P300 timeframe (250–500 ms) recorded in response to Spanish contrasts related to English nonword repetition performance. This study provides further evidence that the P300 ERP in response to native phonemic contrasts indexes PWM skills, specifically nonword repetition performance, in monolingual children. Further work is necessary to determine the extent to which the P300 response to changing phonological stimuli reflects PWM skills in other populations.

## Introduction

Phonological working memory (PWM) has been implicated in vocabulary development (Gray et al., 2022), sentence processing (Montgomery, 1995), and reading development (Baddeley, 2003), yet the exact neural mechanisms associated with PWM remain unknown, particularly within children. The event-related potential (ERP) technique allows for an objective measure of the neural activity associated with PWM due to its excellent measurement of the temporal order of human speech. One highly studied ERP is the P300 component. The P300 is often cited as a measure of attention, however, the P300 may also index other executive processes. Previous research using computational modeling has suggested that the P300 component is an index of PWM (Bonala and Jansen, 2012). To the authors’ knowledge, there have been no studies that have specifically investigated the P300 component and its relationship to clinical measures of PWM in early school-aged children. One particular measure of PWM is nonword repetition. nonword repetition tasks require the repetition of novel words and has been cited as a “pure” measure of PWM given the reliance on memory for speech sounds that are not linked to semantic content. Given that the P300 has been cited as a measure of PWM and nonword repetition has been used for decades as a clinical measurement of PWM, the relationship between the P300 and nonword repetition warrants investigation.

We investigated the neural signature of the P300 response recorded from native-English and non-native-Spanish phonemic contrasts in a sample of 33 typically developing monolingual English-speaking first- and second-grade children. By using both native and non-native speech contrasts we can determine the extent to which the “neural commitment” to the native language impacts the presentation of the P300 response in terms of amplitude and latency parameters. Further, we explored the relationship between the P300 response and clinically relevant behavioral measures of PWM, including both nonword repetition and digit span, to validate the P300 response as a measure of PWM in children.

### Phonological working memory in children

Phonological working memory includes the short-term storage of phoneme information for later manipulation (Wagner and Torgesen, 1987).^1^ One of the most highly studied models of working memory comes from Baddeley and colleagues. The latest version of Baddeley’s model includes four working memory components: the central executive, the phonological loop, the visuospatial sketchpad, and the episodic buffer (Baddeley, 2000). The central executive entails attention to stimuli and regulates other working memory components. The phonological loop allows for the temporary storage and rehearsal of phonological information. The visuospatial sketchpad processes visual and spatial information and the episodic buffer is a storage system that binds information from several sources into a “single multifaceted code” (Baddeley, 2003).

There is a vast literature on the relationship of PWM to the development of oral and written language development (see Baddeley, 2003 for review). In terms of written language, PWM is a specific skill under the umbrella of general phonological processing abilities. Along with phonological awareness and rapid automatized naming, PWM skills are predictive of word reading abilities, particularly within early school-aged children (Wagner and Torgesen, 1987; Lonigan et al., 2000; Hogan et al., 2005). PWM may be used during reading decoding to temporarily store phonological segments during the mapping of graphemes to phonemes and prior to output. Also, poor PWM may impede the development of strong phoneme-grapheme correspondences due to inefficiencies in maintaining sound segments within the phonological loop (Gathercole and Baddeley, 1993). Given its importance in the reading process (as well as oral language), clinical measures of PWM play a critical role in the early identification of language and reading impairments in early school-aged children as they begin the reading process (Conti-Ramsden and Durkin, 2007).

Nonword repetition (NWR) tasks are the field’s “gold standard” measure of PWM, particularly when those tasks contain longer items (Gathercole, 2006). NWR entails listening to the target nonword presented auditorily, holding it in memory for a short time, and then repeating phonological information verbatim following a stimulus; therefore, requiring PWM skills. Nonwords are not linked to semantic content; however, they can vary in terms of length, complexity, and wordlikeness. Given the absence of semantic content, NWR has been cited as a more stringent measure of PWM skills, such that item familiarity (as in the case of digit span) is absent (Gathercole et al., 1994). NWR performance specifically taps core speech perception, linguistic/phonological encoding, storage, and production (Gathercole, 2006), and therefore may recruit neural mechanisms responsible specifically for phonological processing. Children identified with a language and/or reading impairment often perform poorly on NWR tasks when compared to typically developing peers (Dollaghan and Campbell, 1998; Rispens and Baker, 2012), providing further evidence that language and/or literacy impairment may stem in part from PWM deficits.

Phonological working memory entails the storage and recall of phonological units, precipitating the need for intact phonological representations and the keen ability to be able to discriminate between phonemic units. In the developmental literature, the ability to discriminate between speech sounds and develop a “strong neural commitment” to the native language has predicted word learning and eventually reading skills further along the developmental trajectory (see Kuhl et al., 2008 for review; Molfese, 2000). This work has focused on the use of ERPs to measure the neural response to speech and determine the nature in which ERPs relate to behavioral measure of language.

### The event related potential technique as a measure of phonological processing

With advances in neuroscience, we can objectively measure the neural response associated with speech perception and phonological processing skills using the ERP technique. ERPs are neural responses that are time-locked to a stimulus, such as speech. Although few studies to date have directly assessed the relationship among PWM and ERPs in early school-aged children, several studies have investigated the mismatch negativity (MMN) component and its relationship to phonological processing skills and early reading development at large (Bonte et al., 2007; Molfese et al., 2008; Hämäläinen et al., 2013, 2018; Linnavalli et al., 2017; Norton et al., 2021). The MMN component is an ERP thought to reflect the discrimination of change in a stream of repeating sounds (Näätänen, 2003). It is passive in nature and can be elicited without having the participant formally attend to the stimuli. The MMN may provide vital information regarding the neural mechanisms which underlie the lower-level processing of speech information.

Two specific studies recorded the MMN in response to native and non-native speech contrasts to determine its relationship to phonological processing in early school-aged children. Linnavalli et al. (2017) reported that within a large sample (*N* = 70) of monolingual and bilingual Finnish children aged 5–6 years, ERPs in response to changing phonemic stimuli were significantly associated with phonemic processing within that same language (e.g., neural response to Finnish contrasts were related to Finnish phonological awareness skills). Hämäläinen et al. (2018) reported that English-speaking children who showed greater responses to non-native Finnish contrasts demonstrated poorer performance on tests of reading accuracy, fluency, and comprehension. These findings collectively suggest that perceptual sensitivity in the native language indexed within the MMN response may be associated with strong phonological processing skills within that language (Noordenbos et al., 2012; Hakvoort et al., 2016).

### Phonological working memory and the P300 event related potential

The MMN response has been linked to phonological processing in young children; however, phonological processing skills are multifaceted and complex. By investigating other components, such as the P300 response, we may be able to determine how different aspects of phonological processing (such as PWM) index unique and specific neural substrates. The P300 ERP is one of the most studied components within the literature. It occurs as a positive peak ∼300 ms post-stimulus onset and is often elicited in response to a “deviant” or less frequently occurring stimulus compared to a highly repetitive “standard” stimulus. The classic P300 effect requires that a participant actively attend to the deviant stimuli. The “context-updating theory” is a prominent theoretical framework of the underlying cognitive mechanisms associated with the P300. Within this framework, executive functioning processes such as attention allow for the comparison of incoming stimuli to previous mental representations (Donchin, 1981; Donchin and Coles, 1988). Context updating supports that several cognitive processes may be reflected in the P300 response including attention and working memory. Studies have linked the P300 to behavioral measures of attention and working memory widely used in neuropsychological assessments of cognition in children, providing further evidence of the link between the P300 and executive skills (Polich et al., 1983; Polich, 2007; Boucher et al., 2010; Brydges et al., 2014).

Bonala and Jansen (2012) set out to specifically measure the relationship between the P300 and PWM. They designed a computational model that mimics the learning mechanisms associated with the P300 component to determine if PWM was responsible for the P300 effect. The model of working memory was based on Baddeley (2000) account, which consists of a phonological loop and visuospatial sketchpad acting as short-term memory storage for content domain areas, and an episodic buffer which links information between the two. Simulation results of the model supported the P300 effect being elicited from a working memory process in line with Baddeley’s model.

Only a handful of studies have investigated the P300 response as a measure of working memory in children. One study by Evans et al. (2011) showed attenuated P300 responses when demands for both auditory and visual working memory increased in children with specific language impairment (ages 11.9–14.0). This study demonstrated the importance of working memory as a key component to language learning and highlighted how the neural underpinnings of working memory in school-aged children may impact language differences among typical and impaired groups. Other studies have investigated differences in the P300 response among dyslexic and non-dyslexic adults (Fosker and Thierry, 2004) and children (Papagiannopoulou and Lagopoulos, 2017). Some have even used the P300 to monitor growth in phonological processing and reading skills following intervention (Alvarenga Kde et al., 2013; Zygouris et al., 2018), yet no studies have directly investigated the relationship between P300 response to measures of PWM, specifically NWR, in early school-aged children.

However, one study has linked the P300 ERP component as an index of PWM to NWR in a sample of typically developing toddlers and young children. Harwood et al. (2017) measured speech perception skills using ERPs recorded in response to changing nonwords (with different phonological features) in a sample of typically developing toddlers. NWR skills were also measured using a specifically designed Test of Early Nonword Repetition (TENR; Stokes and Klee, 2009) which included the phonemes present in early childhood. The ERP P3a^2^ component was highly correlated with NWR performance, so much so that it was unable to be used as a separate predictor within a regression model to determine its contribution to general language skill within the group due to highly correlated variables. The authors determined that both the P3a and NWR tasks were significantly indexing PWM skills. They concluded that further work was necessary to determine the cognitive processes underlying the P300 component in the pediatric population and its relationship to language and or literacy readiness skills such as phonological processing.

### Current study

The current study investigated the P300 response to native and non-native phonemic contrasts within a typical sample of English-speaking monolingual early school-aged children. By using the native English and non-native Spanish contrasts, we extended previous ERP studies using the MMN response to evaluate the role of neural commitment to native phonology within a new ERP component of interest, the P300. Further, we determined the extent to which phonemic sensitivity to native phonology reflected within the P300 response, is related to PWM in early school-aged children. By determining the relationship between the P300 response and behavioral measures of PWM (such as NWR) we were able to not only validate models of the P300 as a component of PWM, but also provide further evidence that NWR tasks are indexing PWM skills. Given these aims, we asked the following research questions:

1) What is the neural signature of English and Spanish phoneme contrasts within the P300 ERP in early school-aged children?
2) Does the P300 ERP recorded in response to English and Spanish phonemic contrasts explain a unique proportion of variance in behavioral measures of English PWM (i.e., PWM Composite, Nonword Repetition, Memory for Digits) in early school-aged children?

The stimuli for this investigation are modeled after Garcia-Sierra et al. (2016). The phonological contrasts included stop consonants for both the English (/ta/, /pa/) and Spanish (/t̪a/, /d̪a/) stimuli. The stimuli were chosen for the following reasons: (1) the use of stop consonants are commonly used for auditory oddball speech paradigms and contain rapidly changing acoustic information and (2) the need for a non-native contrast that would include the stop articulatory feature, but also remain allophonic to non-native listeners. The Spanish contrasts are allophonic variations of the lingua-alveolar English stop consonants (/ta/, /da/). We hypothesized that English-speaking monolingual children would demonstrate a P300 response to English contrasts; however, they would not discern phonemic differences between the Spanish tokens, and therefore would not demonstrate a P300 response for Spanish contrasts. This would therefore be evidence of neural commitment to native language contrasts within the P300 component. The second aim was to determine the relationship among English and Spanish phonemic contrasts within the P300 ERP and behavioral measures of PWM often used in clinical assessment batteries. Given the previous research, which explores neural commitment to language and subsequent phonological processing abilities, we expected that the P300 effect in response to English phonemic contrasts would significantly predict English PWM skills. Based on data from Hämäläinen et al. (2018), ERPs in response to Spanish contrasts would be inversely related to English phonological processing skills. Essentially, greater phonemic sensitivity to non-native contrasts within the P300 ERP would be related to poorer performance on English measures of PWM.

## Materials and methods

### Participants

A sample of 33 first- and second-grade children between the ages of 6.2–8.7 years (Mage: 7.1 years, 13 females) were recruited from the same public elementary school. First and second graders are at a critical point in the reading process in which phonological processing remains highly predictive of word reading abilities (Wagner et al., 1997). Also, these students were able to participate in the EEG (including sitting still and quiet for extended periods of time) and, therefore, due to these reasons, were chosen as the population of interest. Children met the following criteria to be included in the study: (1) were not receiving special education services, (2) hearing and vision were within normal limits or corrected at the time of the study per educational record, and (3) were considered monolingual English speakers per parent report. Although articulation was not formally assessed within our battery of tests, two licensed speech-language pathologists oversaw all the testing for this study and were able to informally measure speech sound production skills. Based on clinical judgment, none of the students presented with a speech sound disorder. If a residual articulation error was present (e.g., substitution of /w/ for /r/), this was not counted as an error in the nonword repetition scoring, which is consistent with standardized rules for scoring nonword repetition tasks.

### Procedures

All study experimental procedures were approved through the University’s Institutional Review Board and the elementary school’s administration team. Parent consent and child assent were obtained prior to the start of the study. To complete language and literacy testing, children participated in 2–5 sessions lasting 45–60 min each during the school day. Children were tested within a quiet environment with minimal distractions. To complete the experimental portion of the study, each child participated in one 2-hour after-school appointment where EEG and eye-tracking tasks were administered. (The eye-tracking task is outside the scope of the present paper and will not be discussed further). Prior to participation in the EEG tasks, children were provided a snack while they listened to a short “social story” which explained each step of the EEG experiment, including the capping process and the expectations during the experiment. Children were allowed to touch the cap and watch as the cap was put on a stuffed bear to ensure that each child was informed and comfortable with the EEG procedures. Children were provided with breaks as needed. Upon completion of the experiment, children chose a small prize.

#### Behavioral speech and language assessment

Each child participated in an extensive battery of language and literacy assessments. This battery included the following tests, which were administered to all participants: (1) *The Wechsler Abbreviated Scale of Intelligence, Second Edition* (*WASI-2*; Wechsler, 2011), a brief measure of cognitive skill. Block Design and Matrix Reasoning subtests were administered to obtain a Perceptual Reasoning/Non-verbal-IQ Composite. (2) The *Receptive One Word Picture Vocabulary Test–Fourth Edition* (*ROWPVT-4;* Martin and Brownell, 2011), a standardized measure of receptive vocabulary. (3) The *Clinical Evaluation of Language Fundamentals-5th Edition* (CELF-5; Wiig et al., 2013), a global measurement of receptive and expressive language of children. Subtests that form the Core Language Composite were administered. (4) The *Comprehensive Test of Phonological Processing–Second Edition* (*CTOPP-2*; Wagner et al., 2013) was administered as a comprehensive measure of phonological awareness, PWM, and rapid naming skills. Two main subtests make up the PWM composite of the CTOPP-2. These tests are Memory for Digits, which requires a participant to repeat strings of numbers accurately; and Nonword Repetition, which measures the ability to repeat nonwords that grow in length and complexity. Typically developing children were considered to have average language skills based on a Core Language Score of 80 or above on the CELF-5, as well as an average Perceptual Reasoning Index on the WASI-II (see Table 1 for standard score ranges of behavioral measures for all participants).^3^ A subset of protocols for each standardized test (20% of the participants) were rescored by trained research assistants to determine the reliability of scoring. Inter-rater reliability for the test scores is as follows: CELF-5 = 98%, CTOPP-2 = 99%, ROWPVT-4 = 100%, WASI-2 = 100%.

**TABLE 1:** Assessment scores for full sample (*N* = 33).

Variable	Range	Mean (*SD*)
Age in years	6.1–8.7	7.1 (0.7)
WASI 2 PR	75–128	101 (12)
ROWPVT 4 ENG	85–129	112 (10)
CELF 5 ENG	80–120	102 (10)
CTOPP 2 PWM	67–122	101 (12)
CTOPP 2 NWR	4–14	9 (2)
CTOPP 2 MD	5–17	11 (3)
CTOPP 2 PA	77–131	103 (12)
CTOPP 2 RN	88–119	102 (8)

WASI 2 PR, Perceptual Reasoning Index of the Wechsler Abbreviated Scale of Intelligence, Second Edition; ROWPVT 4 ENG, Receptive One Word Picture Vocabulary Test, Fourth Edition, English Version; CELF 5 ENG, Clinical Evaluation of Language Fundamentals, Fifth Edition, English Version; CTOPP 2 PWM, Phonological Working Memory Composite of the Comprehensive Test of Phonological Processing, Second Edition; CTOPP-2 NWR, Nonword Repetition subtests of the Comprehensive Test of Phonological Processing, Second Edition; CTOPP-2 MD, Memory for Digits subtest of the Comprehensive Test of Phonological Processing, Second Edition; CTOPP 2 PA, The Phonological Awareness Composite of the Comprehensive Test of Phonological Processing, Second Edition; CTOPP 2 RN, Rapid Naming Composite of the Comprehensive Test of Phonological Processing Second Edition. Standard scores are presented for the WASI 2 PR, ROWPVT 4 ENG, CELF 5 ENG, CTOPP 2, PWM, CTOPP 2 PA, and CTOPP 2 RN. These scores have a population average mean of 100 and SD of 15. Scaled scores with a mean of 10 and SD of 3 are reported for CTOPP 2 NWR and CTOPP 2 DS.

#### Event related potential procedures

Children were fitted with a 128-sponge Ag/AgCl electrode high-density sensor array net (Magstim/EGI, Inc.) that was used to record electrophysiological data. The net was soaked for 10 min in a warm potassium-chloride (KCl) solution to improve conductance and then fitted on the child’s head using standard procedures outlined by EGI (Dien, 2010). EEG data were recorded using Net Station v. 5.4 software (Magstim/EGI Inc.) with an EGI Net Amps 400 high impedance amplifier, at a sample rate of 1000 Hz. All electrode impedances remained under 40 kΩ as indicated by impedance measures made immediately before and after the test sessions. Children were seated comfortably at a desk and listened to the stimuli through two portable Sony speakers placed six feet in front of them at a comfortable listening level (approximately 65 dB).

##### Event related potential stimuli

The experiment consisted of four blocks (two passive blocks and two active blocks) in which either English or Spanish phonemic contrasts were presented within a classic auditory oddball paradigm. In the English block, syllables were produced by an adult male monolingual English speaker: The syllable /ta/ was presented on 200 standard trials (83%) with a duration of 360 ms and a voice onset time (VOT) of 80 ms, while the syllable /pa/ was presented on 40 deviant trials (17%) with a duration of 383 ms and a VOT of 64 ms. In the Spanish block, syllables were produced by an adult native Spanish-speaking male: The dentalized /t̪a/ was presented on 200 standard trials (83%) and had a VOT of 17 ms, while the dentalized /d̪a/ was presented on 40 deviant trials (17%) and had a VOT of −50 ms. Both Spanish tokens were 305 ms in duration. The experiment was designed so that each deviant token was preceded by at least two standard tokens. There was a varied inter-stimulus interval of 950 or 1150 ms. E-Prime v.2.0 (PST, Inc.) was used to control stimulus presentation and record stimulus onsets in Net Station.

Children were required to listen carefully and actively push a button every time they heard the deviant stimuli. This experiment was performed in the context of a larger study, which included other EEG experiments. Prior to each of the current English and Spanish active blocks, an 11-minute passive block, which included the same stimuli, was played (e.g., the English passive block was always played prior to the English active block and the same for the Spanish blocks). The purpose of the passive block was to collect ERP data for another research question not discussed within this manuscript. During those passive blocks, children sat comfortably and were allowed to watch short, unrelated video clips (which did not include speaking faces) on an iPad. The order of Spanish and English blocks was counterbalanced between participants. The active experiment was 16 min long in total.

##### Event related potential processing

EEG data were analyzed using the EEGLAB v2019.1 (Delorme and Makeig, 2004) and ERPLAB 7.0 (Lopez-Calderon and Luck, 2014) toolboxes. As data from some participants were contaminated with line noise, notch filters were applied at line frequencies (60, 120, 180, and 240 Hz: order = 180). The PREP pipeline is a standardized early-stage pre-processing pipeline that identifies bad channels via multiple methods that take into account channel noise and covariation between channels (Bigdely-Shamlo et al., 2015). This pipeline was used to identify electrodes that were bad throughout data collection; these electrodes were replaced using spherical spline interpolation (Perrin et al., 1989). Independent Components Analysis (Makeig et al., 1996) and the ICLabel algorithm (Pion-Tonachini et al., 2019) were used to automatically identify components that represented ocular movements in the EEG data (blinks and lateral eye movements) with at least 80% probability; these components were removed prior to subsequent analysis. Data were band-pass filtered from 0.3 to 30 Hz (Butterworth filter, 12 db./oct roll-off); re-referenced to the average reference (the vertex reference, Cz, was used during recording); segmented into 900 ms epochs including a 100 ms pre-stimulus baseline and an 800 ms post-stimulus interval; and baseline-corrected using the mean of the pre-stimulus window (Junghöfer et al., 1999). Horizontal electrooculogram (HEOG) was measured as the difference between channels 125 and 128, and vertical electrooculogram (VEOG) was represented by four pairwise differences between channels 126 and 8, 126 and 14, 127 and 21, and 127 and 25. Epochs were marked as containing ocular activity if HEOG activity (max-min) exceeded 55 ms within a sliding 80 ms window (indicating a lateral eye movement) or if VEOG activity in any of the four electrode pairs (max-min) exceeded 140 ms within a sliding 80 ms window (indicating a blink); these epochs were not analyzed further. Subsequently, automated routines marked an electrode as bad in an epoch if its activity (max-min) within that epoch exceeded 200 μV, after which that electrode was interpolated on that epoch using spherical spline interpolation. If an electrode was marked as bad for more than 40% of epochs, data from that electrode were interpolated on all epochs. If a segment contained at least 20 bad electrodes, the segment was marked as bad and excluded from analyses. Finally, artifact-free segments were averaged into standard and deviant conditions for a total of 40 trials for each condition. (To balance trial counts across standard and deviant conditions, only standard trials that immediately preceded deviant trials were included in analyses). A criterion of at least 15% (at least six trials per condition) preserved trials within both conditions (standard and deviant) was used to include participants in the ERP analysis for each language.^4^ Four participants were excluded from English ERP analyses (*N* = 29) and three participants were excluded from Spanish ERP analyses (*N* = 30) due to low usable trial counts. There was no significant difference in the average number of good trials preserved within the English block between conditions (standard condition, *M* = 24.6, *SD* = 10.7, range = 7–40; deviant condition, *M* = 25.2, *SD* = 11.7, range = 6–40; *t*(28) = 0.81, *p* = 0.425) or for the Spanish block (standard condition, *M* = 22.4, *SD* = 7.3, range = 8–40; deviant condition, *M* = 23.5, *SD* = 7.6, range = 8–39; *t*(29) = 1.89, *p* = 0.068) for the sample.

##### Event related potential analysis method

**FIGURE 1:**
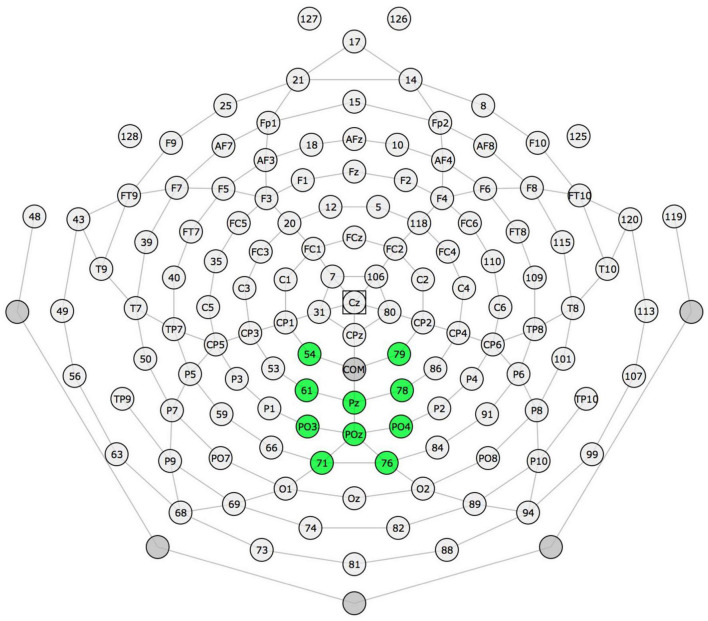
Electrode montage for English and Spanish P300.

The electrode montage (parietal region) and time windows of interest were modified from previous literature on the P300 response in children (Key et al., 2005; Papagiannopoulou and Lagopoulos, 2017). Based on the topographic plot for the 128 electrodes of the grand average (all the participants averaged together for the English and Spanish experiments separately), the data were visually inspected to determine where in the parietal region the P300 was observed. The P300 was identified visually in a cluster of 10 electrodes in the medial-parietal region (see Figure 1 for electrode montage) for the English contrasts (a P300 effect was not observed anywhere in the region for the Spanish contrasts, either at these or at any other parietal electrodes). Electrodes within this cluster were averaged for subsequent analysis, which was performed using the ERPLAB ERP Measurement Tool (Lopez-Calderon and Luck, 2014). Difference waves were generated by subtracting the mean amplitude of the standard from the deviant stimulus (deviant - standard) at each latency. Mean P300 amplitude was computed by averaging across all latencies in the difference wave from 250 to 500 ms post-stimulus onset. Although positivity for the deviant response extends beyond this timeframe, the difference wave which will be used for statistical analyses appears to “peak” within this time window. (We also report descriptive statistics of mean amplitude for the standard and deviant conditions; those mean amplitudes were computed in the same way). To determine fractional peak latency, the most positive local peak that was positive over three samples (6 ms) was identified, after which the 50% fractional peak latency (the time at which voltage reached 50% of this local peak) was computed (Kiesel et al., 2008). For a small number of participants, this latency measure was non-computable due to difference wave morphology (English contrast: *N* = 1; Spanish contrast: *N* = 3); these participants were excluded from latency analyses. These mean amplitude and fractional peak latency of the difference wave were used for regression analyses.

### Statistical analyses

To address Research Question 1, we determined trends in the neural signature of the P300 ERP in response to English and Spanish contrasts using descriptive statistics (mean and standard deviation of amplitude data for both standard and deviant responses as well as the difference wave and mean and standard deviation of latency data for the difference wave; peak identification for latency computations was less well-defined for individual conditions). To address Research Question 2 of whether the P300 ERP within the English experiment and/or the Spanish experiment explained a significant amount of variance within measures of PWM, separate linear regression analyses were conducted to predict behavioral standardized scores (dependent variable) in the areas of PWM on the CTOPP-2 (i.e., PWM Composite, Digit Span, and NWR). The P300 predictor data (independent variable) was the mean amplitude (measured in μV) and fractional peak latency (measured in ms) within the P300 for the difference wave for each language. A Bonferroni correction was conducted to account for the six comparisons within each language; accordingly, a comparison was only deemed significant when *p* < 0.0083 (0.05/6). Statistical analyses were conducted using R (version 4.1.2; R Core Team, 2021).

## Results

Our research questions specifically addressed the relationship between PWM and the P300 response to English and Spanish contrasts. In terms of behavioral performance on working memory tasks of the CTOPP-2, all scaled scores for subtests, including Memory for Digits (*M* = 11, *SD* = 3) and NWR (*M* = 9, *SD* = 2) fell within the Average range. The Phonological Working Memory Composite fell within the Average range (*M* = 101, *SD* = 12). One participant did obtain Below Average scores for all working memory measures. All CTOPP-2 scores were normally distributed, and no outliers were present within the behavioral data.

### English contrasts

**FIGURE 2:**
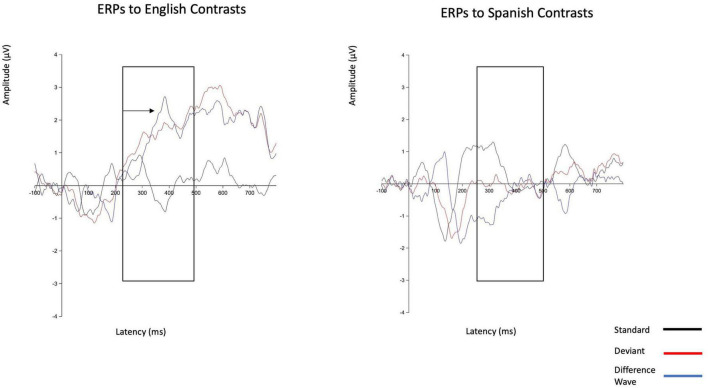
Grand average event related potential Waveforms averaged across the 10-electrode cluster for Monolingual English-speaking children in response to English and Spanish contrasts. The ERP in response to English contrasts demonstrates greater positivity for the deviant response (as is typical for the P300 component). Greater positivity for the deviant response was not generated in response to the Spanish contrasts.

**FIGURE 3:**
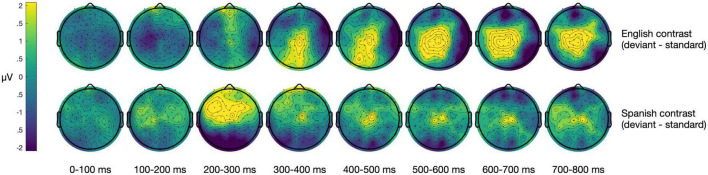
Scalp maps showing the difference wave (deviant minus standard) at all electrodes in 100 ms bins for the English and Spanish contrasts. Significant P300 effects are present for the English contrasts within timeframes of interest. No significant effects are present for the Spanish contrasts within the central-parietal electrodes for the timeframes of interest.

Within the English experiment (*N* = 29), the average P300 amplitude within the standard condition was 0.10 μV (*SD* = 2.18 μV) and within the deviant condition was 1.67 μV (*SD* = 3.37 μV). The average mean amplitude of the difference wave was 1.57 μV (*SD* = 3.80 μV), indicating a statistically significant group-level P300 effect [*t*(28) = 2.23, *p* = 0.034]. The average fractional peak latency of the difference wave was 366 ms (*SD* = 68 ms). See Figure 2 for waveforms and Figure 3 for scalp maps of the difference wave.

Separate linear regressions were conducted to determine the amount of variance explained in behavioral measures of PWM measured by CTOPP-2 tasks (NWR, Memory for Digits, and PWM Composite) from the P300 ERP difference wave recorded in response to English contrasts within the latency and amplitude domains (a total of six statistical tests, corrected as described above). Age was included in each model to account for changes in speech perception due to maturation. All *R*^2^ values reported (both overall and partial) are adjusted.

When predicting NWR performance from the latency difference with the P300 component and age, the model was significant, *F*(2, 25) = 8.29, *p* = 0.002. Within the model, fractional peak latency within the P300 ERP accounted for significant variance in NWR performance (*R*^2^_partial_ = 32%, *B* = −0.02, *p* < 0.001) but age did not (*R*^2^_partial_ = 14%, *B* = −1.04, *p* = 0.030); thus, better NWR performance was associated with earlier peak latencies within the difference wave (i.e., earlier P300 responses). Models predicting Memory for Digits and the PWM Composite from the P300 latency difference were not significant [Memory for Digits: *F*(2,25) = 0.44, *p* = 0.651; PWM Composite: *F*(2,25) = 1.43, *p* = 0.258]. In terms of amplitude data within the P300 component, the difference in mean amplitude between the deviant and standard stimuli did not predict any measure of PWM on the CTOPP-2 (all *F* < 1.72, all *p* > 0.200) (see Table 2 for English regression statistics).

**TABLE 2:** Linear regression models predicting CTOPP-2 performance from English P300 event related potential (*n* = 29).

		Unstandardized coefficients		Standardized coefficients	Predictor significance	Model fit			
						
Model	Variable	*B*	*SE*	β	*P*	*R* ^2^	*F*	*P*	Dependent variable (predicted)
1	Lat Dif	−0.03	0.03	−0.20	0.300	0.03	1.43	0.258	PWM composite
	Age	−4.82	3.32	−0.28	0.160				
2	Lat Dif	−0.02	0.00	−0.57	**0.000**	0.35	8.29	**0.002**	Nonword repetition
	Age	−1.04	0.45	−0.36	0.030				
3	Lat Dif	0.00	0.01	0.12	0.560	−0.04	0.44	0.651	Memory for digits
	Age	−0.52	0.83	−0.12	0.540				
4	Amp Dif	0.64	0.57	0.21	0.270	0.03	1.40	0.264	PWM composite
	Age	−4.59	3.21	−0.27	0.160				
5	Amp Dif	0.12	0.09	0.23	0.220	0.05	1.71	0.200	Nonword repetition
	Age	−0.84	0.53	−0.30	0.130				
6	Amp Dif	0.09	0.14	0.12	0.540	−0.04	0.44	0.650	Memory for digits
4.	Age	−0.64	0.80	−0.16	0.430				

As a Bonferroni correction was applied to control family-wise error rate across six comparisons, *p*-values are significant (and thereby shown in bold) only if they are less than 0.0083 (0.05/6). One participant was removed for correlations involving P300 latency due to not having an identifiable peak for fractional peak latency computations. Lat Dif: Fractional peak latency of the difference wave of the P300 Component. Amp Dif: Mean amplitude of the difference wave of the P300 component. PWM Composite: Phonological Working Memory Composite of the CTOPP-2. Significant values (*p* < 0.0083) are indicated in bold.

### Spanish contrasts

Within the Spanish experiment (*N* = 30), the average P300 amplitude within the standard condition was 0.40 μV (*SD* = 2.72 μV) and within the deviant condition was −0.08 μV (*SD* = 2.62 μV). The average mean amplitude of the difference wave was −0.47 μV (*SD* = 3.83 μV). The deviant trials were on average *less* positive than the standard trials within the 250–500 ms time window, indicating that the group did not demonstrate a P300 effect for Spanish contrasts, *t*(29) = −0.68, *p* = 0.503. The average fractional peak latency of the difference wave was 377 ms (*SD* = 59 ms).

Separate linear regressions were conducted to determine the amount of variance explained within all PWM CTOPP-2 from the Spanish ERP data within the P300 timeframe (250–500 ms) within the latency and amplitude domains (six statistical tests, corrected as above). None of the models were statistically significant (all | *F* < 2.74, all *p* > 0.085), suggesting that the phonemic discrimination to non-native contrasts measured within the P300 timeframe did not explain significant variance within English PWM (see Supplementary Table 1 for Spanish regression statistics).

## Discussion

The current study investigated the neural indices of speech perception to native and non-native speech contrasts within the P300 ERP and its relationship to measures of phonological working memory (PWM) in monolingual English-speaking early school-aged children. Our first research question was to determine the neural signature of English and Spanish phoneme contrasts in monolingual English speaking early school-aged children. Given that the English contrasts were part of the native phonology, and the Spanish contrasts were allophonic, we expected to see distinct differences within the P300 response. The children within this study demonstrated a P300 response within the standard timeframe (250–500 ms) for English contrasts but failed to show a P300 effect for Spanish contrasts throughout the ERP epoch. This was expected as the Spanish tokens were allophonic to native English speakers and therefore, the P300 responses did not discriminate between the dentalized /t̪a/ and /d̪a/ contrasts.^5^

To address research question 2, the ERP data in response to English and Spanish contrasts was further investigated to determine its relationship to English measures of PWM. The fractional peak latency of the difference between the standard and deviant P300 response for English contrasts significantly predicted nonword repetition (NWR) standard scores on the CTOPP-2. The estimated slope in the regression was negative, indicating that children with higher NWR scores showed sensitivity to the contrast (deviant vs. standard stimuli) earlier than children with lower NWR scores. There was not a significant relationship between P300 ERPs recorded to Spanish contrasts and PWM skills. Implications of these results are discussed in detail below.

### P300 response and phonological working memory

To the authors’ knowledge this is the first paper to report a direct relationship between the P300 ERP response and NWR performance in a sample of typically developing school-aged children. Given that the P300 has been cited as an index of PWM within a computational study (Bonala and Jansen, 2012) this relationship between the P300 and NWR validates NWR as a clinical measure of PWM. Further, these results are also consistent with the P3a effect relating to NWR skills within young children. Harwood et al. (2017) found a strong relationship between NWR performance on the TENR (Stokes and Klee, 2009) in a group of typically developing children aged 2–3 and the P3a ERP in response to similar sounding nonwords. In that study, faster latencies were correlated with higher language performance. This relationship between P300 and specifically NWR in children warrants further study, as PWM is a critical cognitive process that has been implicated in several aspects of oral and written language development in children.

Our results indicated that latency measures were predictive of NWR performance; however, amplitude measures were not significant. P300 latency has been reported to measure classification speed, which may be proportional to the time required to detect and evaluate a target stimulus (Kutas et al., 1977; Magliero et al., 1984). Further, individual differences for P300 latency are correlated with processing speed such that shorter latencies are associated with higher performance on cognitive assessments (Pelosi et al., 1992). It is possible that stimulus classification is critical for NWR performance. Further, latency values in general may be associated with myelination and synaptic efficiency (Eggermont, 1988; Ponton et al., 2000) while amplitude measures may reflect growth of synaptic density, improved synaptic efficiency, and spatio-temporal synchronization (Choudhury and Benasich, 2011). Earlier latencies for novel phonological stimuli may signal effective neural networks, specifically growth within white myelin tracts, that are distinctly used for phonological processing.

Attentional processes have also been implicated within the P300 response. It is possible that the P300 ERP within this experiment is particularly indexing attention to differences between salient phonological features between native contrasts. Astute attention to phonological structure is also required for NWR performance. Also, components of Baddeley’s model of working memory entail attentional resources (i.e., the central executive). Therefore, it is not possible at this time to clearly identify which components of PWM are being reflected in the P300 response. However, the use the auditory oddball paradigm with phonemic contrasts may specifically recruit neural mechanisms associated with attention to the phonological structure and one’s capacity to hold phonological information in short term memory for comparison (i.e., the phonological loop). Therefore, the unique properties of this paradigm should be considered for future research when investigating the P300 response and PWM.

Interestingly, the P300 ERP predicted NWR skills; however, it failed to predict digit span. This finding is inconsistent with previous studies that have correlated P300 response to digit span measures (Polich et al., 1983). Although digit span is often used as a measure of working memory in clinical batteries of language and cognition, some researchers have failed to determine a strong correlation between span tasks (digit span, letter span, and word span) and measures of language (Perfetti and Goldman, 1976). It is possible that span tasks tap into aspects of working memory as a passive storage buffer but do not sensitively measure other aspects of processing as well as storage capacities (Daneman and Merikle, 1996). Span tasks often include the recall of stimuli that is linked to semantic aspects such as the names of letters and numbers. NWR is specifically designed to tap PWM processes in the absence of semantic restraints. The results of our findings may suggest that the P300 response to changing phonological stimuli may be more strongly related to NWR tasks which recruit PWM and/or specific attentional capacities mainly associated with phonological structure.

It was predicted that children would not demonstrate a P300 effect to Spanish phoneme contrasts (given that they are allophonic contrasts in English) and that sensitivity to such a phoneme contrast within the P300 might even be inversely related to PWM performance (with larger or earlier P300 responses being negatively related to NWR performance). However, analyses did not reveal an inverse effect. The grand average for the group revealed that the ERP response to the standard contrast was numerically more positive than the deviant condition (in contrast to the typical P300 effect), and there was no relationship between P300 ERPs to Spanish contrasts and English phonological processing skills. Although an inverse relationship between the P300 and PWM scores was not present within the current study (which could be due to several factors such as characteristics of the components measured, the nature of the stimuli, ERP analysis methods, the sample characteristics, and the overlap between the phonological properties of the languages studied), our current results reflect some similar conclusions. The children who demonstrated earlier sensitivity to English native contrasts within the P300 demonstrated greater skill in tasks of PWM, namely, NWR.

### Clinical implications

Phonological processing skills play a significant role in reading development, and assessment of phonological processing capabilities are common clinical practice. The current study reveals that the P300 ERP in response to English contrasts is significantly associated with English PWM tasks, namely NWR for children. It is possible that the P300 ERP is a measure of the neural mechanisms associated with NWR. Demonstrating a relationship between ERPs and clinical assessment batteries for children is the critical first step in determining the clinical utility of ERPs. Several stages of standardization would be required to determine if ERPs had the potential to demonstrate the levels of diagnostic accuracy necessary for clinical use. Although still in the experimental stage, with advances in technology and brain sciences, electrophysiological markers of cognition may 1 day provide additional clinical information to comprehensive batteries of behavioral assessments. This information may potentially guide clinical decision-making regarding identification of children who may present atypical processing capacities and therefore require services (Näätänen, 2003). Further exploration of the neurobiological markers associated with behavioral NWR performance can provide meaningful evidence to theories on the cognitive processes underlying NWR performance.

The current results have implications for clinicians working with children. In terms of assessment, NWR tasks can play an important role as part of a comprehensive assessment battery for children suspected of language impairment. NWR can provide important insights into PWM capacities and possible limitations. Recent work suggests that children with dyslexia have a specific deficit in the phonological and central executive working memory factors when compared to typical peers (Alt et al., 2021). This work should be extended to clinical populations to determine how children with language and/or reading impairment may differ in terms of their P300 recordings to phonological contrasts when compared to children with typical skill. In terms of intervention, by supporting phonological development from a young age, clinicians can enable keen perception skills and eventually strong phonological processing abilities necessary for reading success. Our findings suggest that sensitivity to the phonological structure of one’s native language reflected within the P300 response is predictive of NWR performance. Therefore, these results align with instructional practices which require children to attend to and manipulate phonological structure. Attention to phonological structure and performing tasks which require children to explicitly discriminate between sounds may support the development of strong phonological processing skills and eventually, strong phoneme-grapheme correspondence.

### Limitations

These findings should be considered in the context of various limitations. First, the sample was relatively small. Further research should be conducted regarding the P300 and measures of NWR to ensure generalization of the results. Additionally, children in this study were considered typically developing and therefore, were not receiving special education services for an identified disability. To the authors’ knowledge, none of the children were identified as having ADHD/ADD or required modifications/accommodations (including a 504 plan) to address attention deficits in schools. However, it is possible that some children may have subclinical attention deficits that may have impacted performance on behavioral assessments or the experimental task.

Acoustic differences within the stimuli make it difficult to discern the exact mechanisms responsible for the elicitation of the P300 response. The native English speech contrast (/ta/, /pa/) is a contrast of the placement feature of speech (/p/ is a bilabial voiceless stop whereas /t/ is a lingua-alveolar voiceless stop). The Spanish contrast presents similar placement and manner features in terms of articulation (both dentalized stops); however, the contrast lies in the voicing differences between the phonemes (/t̪a/ voiceless, /d̪a/ voiced). Therefore, the contrasts are not consistent between native and non-native pairs in terms of place or voicing contrasts. It is possible that the greater acoustic difference between the native English contrasts (with formant transitions being different across a long portion of the syllable) led to the P300 effect. Further research is necessary to determine how differences in the salient features of the acoustic stimuli contribute to the P300 mechanism as well as how attention to native and non-native contrasts are reflected within the P300 response. Also, in terms of accuracy accounts, although children were instructed to use the button press during the experimental task, button press accuracy data was not collected; therefore, behavioral accuracy levels for discrimination between the English and Spanish phonemic contrasts is not available. Further limitations include data loss due to artifacts, which is common in ERP studies with children. Although great measures were taken to remove artifacts and preserve clean trial counts, some children presented low trial counts.

### Future directions

As mentioned above, future investigations of the P300 response in young children should include both typically developing and language impaired and/or reading impaired groups. By using well defined groups, we may be able to stratify subtle differences in working memory that may uniquely contribute to specific language and or literacy profiles that often have overlapping behavioral features. Differences between the P300 response between typically developing and clinical populations may reveal subtle, yet important differences about the neurobiological bases of impairment in children.

Further, although the focus of the investigation included monolingual English-speaking children, continued investigations which explore the relationship between early speech perception abilities and phonological processing skills in bilingual children, specifically Spanish-English bilingual learners within the US are warranted. Spanish-English bilingual children make up the greatest proportion of English Language Learners in America (National Center for Education Statistics [NCES], 2021). By examining neurocognitive features of language and literacy in bilingual children, we can evaluate subtle, yet critical differences in the neurocognitive learning mechanisms associated with bilingualism that may not be evident with behavioral testing. Further research is necessary to determine relationships among PWM within the P300 ERP and measures of language for Spanish-English bilingual children. The inclusion of Spanish-English bilingual children in future studies of speech perception and phonological processing is critical for determining how exposure to two languages shapes phonological development and how phonological processing impacts reading performance. This has both theoretical and clinical implications to educators and clinicians who support, assess, and treat this important population of students.

## General conclusion

Phonological working memory is highly implicated in language and literacy development. The P300 ERP has been cited as an index of PWM, but little is known regarding its relationship to behavioral measures of PWM in children. The current study provides evidence that the P300 response recorded from native English phonemic contrasts significantly predicted a highly used measure of PWM, namely NWR in monolingual English-speaking early school-aged children. This finding validates the P300 and NWR as measures of PWM. In terms of the Spanish contrasts, children did not elicit a P300 response to Spanish allophonic contrasts, nor was ERP data from Spanish contrasts related to PWM skills. These findings are consistent with other ERP studies demonstrating that a strong commitment to the native phonology relates to language performance in children. Future studies are needed to: (1) determine the nature among the P300 response and PWM skills in children and (2) determine how neural markers of English and Spanish speech sounds relate to PWM skills within the P300 ERP for both English-speaking monolingual and Spanish-English bilingual students.

## Data availability statement

The raw data supporting the conclusions of this article will be made available by the authors, without undue reservation.

## Ethics statement

The studies involving human participants were reviewed and approved by URI Institutional Review Board. Written informed consent to participate in this study was provided by the participants’ legal guardian/next of kin.

## Author contributions

VH was responsible for the conceptualization, experimental design, data collection, data analyses and manuscript writing for this project. AB was responsible for the conceptualization, data collection, and manuscript writing. DK was responsible for EEG data processing and analyses. GP was responsible for statistical consultation and analyses. All authors contributed to the article and approved the submitted version.

## Footnotes

1 The authors recognize that several differentiating terms (i.e., verbal working memory, short term memory, PWM) are used within the literature, and therefore, for the remainder of this paper, we will use PWM.

2 The P3a component has a frontocentral scalp topography. Similar to the classic P300 response, P3a is elicited by the presentation of a unique or novel stimuli in a train of repeated standard targets.

3 One participant obtained a Below Average score on the WASI-2 Perceptual Reasoning Index; however, all language scores for this participant fell within the Average range and was therefore included in the sample.

4 The criteria of 15% for accepted ERP trials was determined *a priori* to data analysis.

5 The scalp maps revealed a P300 response for the English contrasts, but not Spanish contrasts, within the electrodes montage (central parietal) and timeframes (250–500 ms) of interest for this investigation. Scalp maps did reveal a significant positivity for Spanish deviant stimuli within an earlier timeframe (200–300 ms) in frontocentral electrodes that diminishes and therefore does not impact our current findings. Further research is needed to determine how neural commitment to native contrasts within different ERP components are related to early reading for this population.
