# Supplementary material for: The P300 event related potential predicts phonological working memory skills in school-aged children
Source: Front Psychol. 2022 Oct 12;13:918046. doi: 10.3389/fpsyg.2022.918046 (PMC9599408; doi:10.3389/fpsyg.2022.918046)
Supplement: Supplementary file 1 [file Table_1.docx]

Supplemental Table 1: Linear regression models predicting CTOPP-2 performance from Spanish ERPs in timeframe (250-500ms) (*n*=30)

|  |  | Unstandardized  Coefficients | | Standardized Predictor  Coefficients Significance | | Model Fit | | |  |
| --- | --- | --- | --- | --- | --- | --- | --- | --- | --- |
| Model | Variable | *B* | *SE* | β | *p* | R^2^ | F | *p* | Dependent Variable  (Predicted) |
| 1. | Lat Dif | .01 | .05 | .03 | .89 | .11 | 1.60 | .22 | PWM Composite |
|  | Age | -.48 | .27 | -.32 | .09 |  |  |  |  |
| 2. | Lat Dif | .01 | .01 | .16 | .38 | .17 | 2.78 | .08 | Nonword Repetition |
|  | Age | -.1 | .05 | -.37 | .05 |  |  |  |  |
| 3. | Lat Dif | -.01 | .01 | -.09 | .64 | .04 | .55 | .58 | Memory for Digits |
|  | Age | -.06 | .07 | -.18 | .34 |  |  |  |  |
| 4. | Amp Dif | -.21 | .59 | -.07 | .72 | .11 | 1.66 | .21 | PWM Composite |
|  | Age | -.46 | .29 | -.31 | .12 |  |  |  |  |
| 5. | Amp Dif | -.00 | .10 | -.00 | .99 | .15 | 2.31 | .12 | Nonword Repetition |
|  | Age | -.10 | .05 | -.38 | .05 |  |  |  |  |
| 6. | Amp Dif | -.07 | .14 | -.11 | .60 | .04 | .59 | .56 | Memory for Digits |
|  | Age | -.05 | .07 | -.15 | .46 |  |  |  |  |

Note: Lat Dif: Fractional peak latency of the difference wave of the P300 Component. Amp Dif: Mean amplitude of the difference wave of the P300 component.  PWM Composite: Phonological Working Memory Composite of the CTOPP-2.
